# Tracing microbial hazards in the aquatic supply chain: challenges, technologies, and future directions

**DOI:** 10.3389/fnut.2025.1673037

**Published:** 2025-10-01

**Authors:** Jiayi Zhang, Tian Ding, Juhee Ahn, Zhaohuan Zhang, Xinyu Liao

**Affiliations:** ^1^Future Food Laboratory, Innovation Center of Yangtze River Delta, Zhejiang University, Jiaxing, Zhejiang, China; ^2^Department of Food Science and Nutrition, Zhejiang University, Hangzhou, Zhejiang, China; ^3^Zhejiang Key Laboratory of Agri-food Resources and High-value Utilization, Zhejiang University, Hangzhou, Zhejiang, China; ^4^College of Food Science and Technology, Shanghai Ocean University, Shanghai, China

**Keywords:** aquatic supply chain, food safety, microbial contamination, traceability technology, artificial intelligence

## Abstract

Aquatic products are a crucial source of dietary protein, especially in regions with abundant marine resources. However, with the expansion of global trade, the risk of microbial contamination in these products has increased, leading to serious public health concerns due to extended transportation and varying regulatory standards. Foodborne illnesses associated with aquatic products not only impact consumer health but also result in significant economic losses due to reduced market confidence, brand damage, and costly recalls. This review systematically examines the role of traceability technologies in enhancing microbial safety in aquatic products. Emphasis is placed on the integration of genome sequencing, artificial intelligence, and digital monitoring systems within the traceability framework. The evaluation considers specific performance indicators, including detection sensitivity (for example, the minimum limit of detection for target pathogens), source attribution resolution (for example, ≤20 core-genome SNP differences or unique wgMLST allelic profiles), and time-to-result in outbreak scenarios, as well as accessibility for small-scale producers and scalability across diverse aquaculture environments. In particular, we outline how artificial intelligence can be integrated with genome sequencing. For instance, WGS-derived genomic fingerprints can be transformed into machine learning models for rapid and highly sensitive microbial source prediction, thereby enhancing real-time decision-making capability along the aquatic product supply chain. Traceability systems have proven effective in enabling real-time monitoring and rapid response to contamination events. Technologies such as genome sequencing and AI significantly enhance detection speed and accuracy, contributing to improved food safety management. Nonetheless, challenges remain, including technological barriers for small-scale producers, fragmented international standards, and low public awareness. To overcome these limitations, future efforts should focus on developing cost-effective and user-friendly traceability tools, promoting global standardization, strengthening regulatory frameworks, and increasing public engagement. Furthermore, innovative approaches involving big data analytics, and AI hold great promise for advancing microbial safety and ensuring the integrity of aquatic product supply chains.

## 1 Introduction

**Figure 1:**
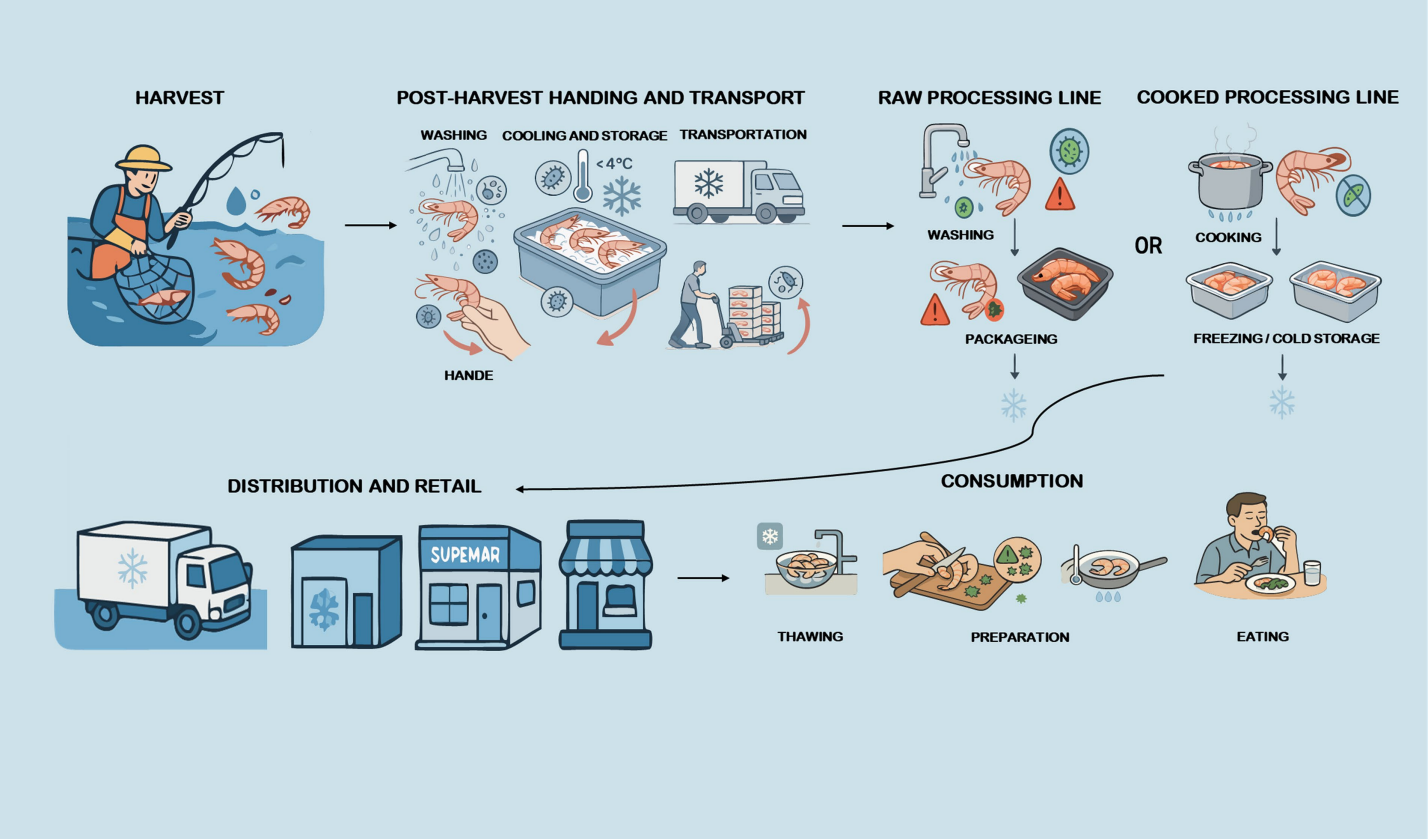
The full supply chain process of aquatic products.

Aquatic products are rich in proteins, omega-3 fatty acids, vitamins and minerals, with good nutritional value, and they have become an important part of the global food market (1). According to the Food and Agriculture Organization of the United Nations, global per capita aquatic product consumption was 20.6 kg in 2021, more than double the average of 9.9 kg in the 1960s (2). Despite their nutritional benefits, aquatic products are uniquely susceptible to contamination by foodborne microorganisms along the supply chain (e.g., aquaculture, processing, distribution, retail, consumption), posing significant public health risks (Figure 1) (3). Common foodborne microorganisms in aquatic products include *Salmonella*, *Vibrio parahaemolyticus*, *Vibrio vulnificus*, norovirus, hepatitis A virus, etc. (4). These microorganisms pose significant public health risks due to their potential transmission from aquatic products to humans. For example, whole genome sequencing analyses have revealed high genomic similarity between isolates from aquatic foods and those from clinical cases, indicating possible direct transmission routes (5).

To mitigate these public health risks and interrupt the transmission chain, effective traceability systems are imperative for assuring the microbial safety of aquatic food chains (6). However, aquatic supply chains face numerous challenges, including fragmented production sites, variable environmental conditions (e.g., water temperature, salinity), and high perishability (7). Traditional approaches, which rely heavily on laboratory culture plate counts for microorganism detection, suffer from slow identification and response times, leading to delayed responses during outbreaks (8). Instead, proactive end-to-end traceability enables real-time monitoring of critical control points (e.g., harvest zones, processing facilities, and logistics), empowering stakeholders to identify, isolate, and mitigate contamination risks before products reach consumers (9).

In this review, we emphasize advancements and gaps in addressing microbial hazards across aquatic supply chains. We evaluated the emerging tools for their efficacy in microorganism tracking and supply chain digitization, and discussed barriers to implementation. By bridging interdisciplinary insights from microbiology, data science, and supply chain governance, this work aims to inform policymakers, industry leaders, and researchers in advancing safer and more transparent aquatic food systems.

## 2 Microbial hazards in aquatic products

### 2.1 *Vibrio* spp.

*Vibrio* spp. are a group of Gram-negative halophilic bacteria, widely distributed in the global ocean, estuarine and coastal waters (10, 11). *Vibrio* spp. exhibit the preference for salty environments and demonstrate facultative anaerobic capacities, enabling survival with or without oxygen (12). In an investigation by Ma et al. (13), it is reported that global prevalence of *V. cholerae*, *V. parahaemolyticus* and *V. vulnificus* in fish was 9.56, 24.77, and 5.29%, respectively. Aquatic products (e.g., shrimps, mussels, oysters) are the potential carriers of *Vibrio* spp. (14). Common *Vibrio* species detected in the aquatic foods include *Vibrio cholerae*, *Vibrio parahaemolyticus*, and *Vibrio vulnificus* (15). Epidemiological data from Australia indicates that a level of 97% of *V. parahaemolyticus*-associated enteric infection outbreaks nationwide were attributed by the consumption of oysters (16).

Cultural methods (e.g., ISO 21872-1:2017) with the use of Thiosulfate citrate bile salts sucrose (TCBS) medium remain as the gold standard for the detection of *Vibrio* spp. in the aquatic foods (Supplementary Table 1). Additionally, the molecular approaches (e.g., PCR, qPCR) are also widely employed for detecting the specific genes: *ctxA* gene for *V. cholerae*, *ldh*, *tlh*, *tdh* and *trh* gene for *V. parahaemolyticus, vvh* gene for *V. vulnificus* (17). However, PCR is unable to discriminate between non-viable and viable cells, potentially leading to overestimate of contamination loads. To overcome this constraint, qPCR approaches incorporating the viability-selective dyes, such as propidium monoazide (PMA) and ethidium monoazide (EMA), have been applied for the detection of total viable microorganisms (18).

### 2.2 *Salmonella* spp.

*Salmonella* spp., a facultative anaerobic gram-negative foodborne microorganism, often causes human infections through contaminated food (19). This bacterium can survive under low temperature and high salty condition, rendering the possibility of their persistence in aquatic foods (Supplementary Table 2) (20). According to the White-Kauffmann classification scheme, *Salmonella* spp. are classified into two species, *S. bongori* and *S. enterica*. This classification is based on the antigenic variations in the surface structures: lipopolysaccharide (LPS), flagella, and capsular polysaccharide. *S. enterica* includes six subspecies, *enterica*, *salamae*, *arizonae*, *diarizonae*, *houtenae*, and *indica*, with over 2,600 serovars (21). In aquatic products (such as fish, shellfish, shrimp, mollusks, etc.), *Salmonella* contamination can result from aquaculture waters (such as water contaminated with faeces or sewage), contaminated feed, cross-contamination during processing, or improper transportation and storage conditions (22). Salmonellosis is prone to lead to fever, diarrhea, vomiting and other gastroenteritis symptoms, especially in children, the elderly and immunocompromised people (23). In the study by Ferrari et al. (24), it is revealed that global *Salmonella*-related foodborne outbreaks related to fish consumption reach up to 12%. The study further demonstrated substantial geographic variation in *Salmonella* serovars distribution across aquatic food products. For instance, in Africa, *S. enteritidis*, *S.* Hadar, *S.* Kentucky and S. Blockley were found to be the most abundant serovars in aquatic food, whereas *S.* Newport emerged as the primary serovar in North America.

### 2.3 Listeria monocytogenes

*Listeria monocytogenes*, a resilient foodborne microorganism, poses a significant microbial hazard in aquatic food products (e.g., shellfish, fish) (Supplementary Table 3) (25). The consumption of food contaminated by *L. monocytogenes* could cause listeriosis with a range of symptoms including fever, nausea, vomiting, diarrhea, headache, etc. (25). The disease is especially dangerous for vulnerable populations including pregnant women, newborns, the elderly, and immunocompromised individuals, potentially causing septicemia, meningitis, or fetal loss (26). *L. monocytogenes* is highly tolerant to cold, high salt concentrations, and acidic environments, allowing it to survive and even multiply under refrigeration, making it particularly difficult to eliminate in aquatic food chains (27). Studies suggest that contamination sources for *L. monocytogenes* in aquatic foods may include cross-contamination during processing, environmental exposure, or contact with contaminated ice used for refrigeration (28). It is estimated that the global incidence of *Listeria monocytogenes* in aquatic foods reached 11% (29).

### 2.4 *Pseudomonas* spp.

*Pseudomonas* spp. are gram-negative, psychrophilic, facultative anaerobic bacteria, one of the most common spoiling microorganisms in aquatic foods, such as fish, shellfish, crustaceans and refrigerated processed products (Supplementary Table 4) (30). Although *Pseudomonas* spp. do not often directly cause foodborne diseases, as a typical spoilage bacterium, it can lead to rapid spoilage of aquatic products by decomposing proteins and lipids, significantly shortening the shelf life, and may indirectly affect food safety (31).

### 2.5 Norovirus

Norovirus is a common cause of gastroenteritis and is widely found in the natural environment (Supplementary Table 5) (32). Noroviruses are non-enveloped viruses with a single-stranded positive-stranded RNA genome, which belongs to the family Caliciviridae (33). HuNoVs can be divided into the genogroups of GI-GX. They are spread through contaminated food, water or contact. Aquatic products are the potential carriers of norovirus (34). Particularly during aquaculture and harvesting, viruses can enter aquatic products through contaminated water sources or unsanitary handling, increasing the risk of human infection. In the study by Li et al. (35), the prevalence of human noroviruses in shellfish was detected as 29% around the world. Symptoms of norovirus infection include nausea, vomiting, diarrhea and abdominal pain, and while usually self-limiting, the health risks are greater for the elderly, the immunocompromised and children (36).

### 2.6 Hepatitis viruses

Hepatitis viruses, particularly hepatitis A (HAV) and hepatitis E (HEV), are significant microorganisms transmitted through the fecal-oral route (37). Both HAV (the *Picornaviridae* family) and HEV (the *Hepeviridae* family) are small, non-enveloped RNA viruses (38). HAV is classified into six genotypes (I, II, III, IV, V, VI) and HEV mainly contains eight genotypes (39). Aquatic products, especially shellfish such as oysters, clams, and mussels, may accumulate these viruses if harvested from contaminated waters or cross-contamination during distribution, processing or retail (Supplementary Table 6). According to a comprehensive literature review, foodborne transmission of HAV is frequently associated with contaminated shellfish and fresh produce, while HEV is increasingly linked to undercooked pork products (40). Acute hepatitis caused by HAV or HEV infection typically presents with symptoms such as fatigue, loss of appetite, nausea, vomiting, and jaundice (38). It is important to note that conventional cooking methods may not always completely inactivate these viruses, particularly HEV (41). It was found that it was only 50% inactivated at 56 °C for 1 h and 96% at 60 °C for 1 h (41).

## 3 Contamination points along the supply chain of aquatic products

### 3.1 Aquaculture

Water is one of the potential microbial contamination sources in aquaculture (42). The quality of aquaculture water (e.g., microbial contamination levels, water temperature, salinity) contributes to the microbial contamination of aquatic products. For instance, Flannery et al. (43) investigated the norovirus contamination of oysters cultivated at a wastewater treatment plant (WWTP) outfall, where oysters were initially free from microbial contamination. Their findings revealed a significant correlation between norovirus levels in oysters and concentrations in effluent wastewater, highlighting water as a critical vector for contamination. Similarly, a study in the coastal oyster breeding farms and fishing ports detected adenovirus (AdVs) and norovirus (NoVs) in water samples at rates up to 40.6 and 11.1%, respectively (44). These viruses, linked to direct discharges of domestic sewage, livestock wastewater, and fishing market effluents into coastal waters, were bioaccumulated by shellfish, posing significant food safety risks. Notably, lower water temperatures were associated with higher detection rates of AdVs and NoVs, particularly during winter months, likely due to enhanced viral stability in colder conditions. Additionally, in the study by Correia Peres Costa et al. (45), it was found that the contamination of lactic acid bacteria, aerobic mesophilic bacteria, *Enterobacteriaceae*, and total coliforms in fish was highly associated with the microbial levels of harvesting water.

Besides aquaculture water, feed is also an important source of contamination, especially when it is improperly stored or comes from unqualified suppliers and can carry microorganisms (46). Furthermore, a study conducted in Ghana indicates that *Escherichia coli*, *Acinetobacter* spp., and *Citrobacter* spp. are frequently detected in fish feed, which can cause diseases in aquatic animals and affect human food safety through the food chain (47). Furthermore, a study conducted in Ghana found a significant positive correlation between the prevalence of bacterial pathogens in the feed and their detection rate in diseased fish tissues (46). Therefore, in the full-chain traceability of aquatic products, tracking and controlling microorganisms in feed are crucial steps to ensure aquatic product safety.

### 3.2 Processing

Cross-contamination is a common risk in the processing of aquatic products. Common sources of contamination during processing include direct contact surfaces for aquatic products, non-direct contact surfaces, processing personnel, and physical/chemical treatment stages. When raw food and cooked food processing facilities are not strictly separated, or the tools and equipment used are not cleaned and disinfected in a timely manner, microorganisms can easily spread from raw water products to cooked food or other foods (48). According to Svanevik et al. (49), pre-capture analysis of fishing vessels revealed contamination of sift boxes, sorting chambers, and pipelines, likely due to exposure to seawater or seabird droppings, with *Escherichia coli* detected in the water samples of refrigerated seawater tank before and after capture, causing fish cross-contamination from poor-quality seawater near sewage outlets. It is also found that over 68% of factory water samples tested positive for enterococci (0.6–2.2 log CFU/100 mL), with landing tanks, washing tanks, and sorting machines exceeding 1.8 log CFU/100 mL. *Listeria monocytogenes* was found in fish and contact points (fishing gear, RSW tanks, conveyor belts, and water samples), indicating potential contamination transfer from vessels to factories. In addition, Møretrø et al. (48) found that industrially processed salmon fillets exhibited higher levels of *Pseudomonas* compared to manually gutted/sliced fish. The dominant *Pseudomonas* sequence types (based on partial 16S rRNA gene) in fish fillets were also prevalent in isolates from equipment post-cleaning, indicating a net transfer of *Pseudomonas* from processing equipment to fillets. These strains colonizing factories likely originate from live salmon or seawater. *Pseudomonas* species are well-adapted to food processing environments, dominating in factories even after cleaning, due to their ability to grow at low temperatures, minimal growth requirements, resistance to bactericidal agents, and propensity to form biofilms, enhancing their survival and colonization in processing plants. Therefore, maintaining good hygiene in processing plants is crucial to ensure the microbiological safety of aquatic food.

In addition to food contact surfaces, non-food-contact areas such as walls, floors, drainage systems, and sewers in the factories can also serve as sources of microbial contamination for aquatic foods during processing (50). Berrang and Frank (51) demonstrated that when a high-pressure water jet strikes the drain wall, residual *Listeria innocua* cells are instantaneously aerosolized into the facility’s air, where those bacteria can disperse horizontally up to 4 meters and vertically up to 2.4 meters. These microscopic droplets disperse through turbulent mixing in the air and then, under the combined effects of gravity and aerodynamic forces, rapidly settle onto equipment, worktables, and exposed seafood, driving cross-contamination. Aerosol generation is driven by the shear forces from the water impact dislodging droplets from the biofilm layer on the drain surface; notably, even in the absence of standing water, a residual biofilm alone can produce substantial aerosols upon impact. While most droplets settle within minutes, this brief period is sufficient for extensive dispersion and deposition. Therefore, during cleaning operations, direct high-pressure spraying of drains or outlets should be avoided, and integrated measures such as optimized drain design, controlled ventilation and routine monitoring and sanitation should be implemented to minimize the risk of aerosol-mediated cross-contamination. Furthermore, temperature control during processing is also crucial. Improper temperature treatment (such as cold chain breaks or substandard processing temperatures) can cause microorganisms to survive and grow on aquatic products. A study on the impact of changes in cold chain conditions for fish pointed out that improper temperature control significantly affects the microbial composition and greatly reduces the quality and shelf life of the fish meat (52).

### 3.3 Distribution/retail

**Figure 2:**
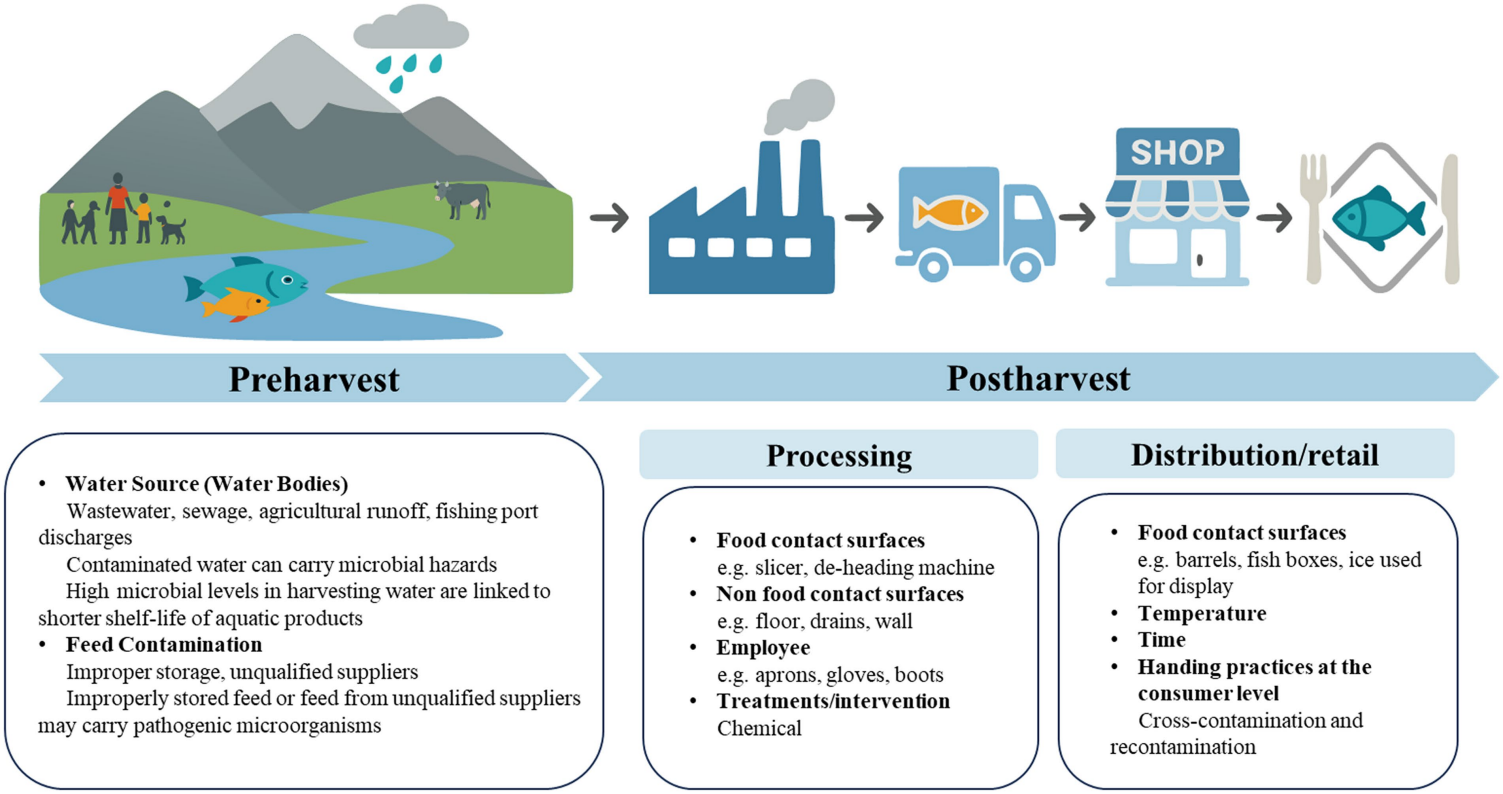
Critical contamination points in the aquatic supply chain.

Distribution and retail are also one of the major points highly associated with the microbial contamination along the aquatic supply chain (53) (Figure 2; Table 1). Cold chain transportation is crucial for maintaining the freshness of aquatic products, and if the cold chain is disrupted during transportation or storage, it can cause temperature rises, promoting microbial growth (54). European Food Safety Authority highlighted that transport conditions critically influenced microbial contamination risk in aquatic products. Under abuse scenarios (e.g., temperature fluctuations or delayed icing), fish tubs (triple-layer polyethylene containers with freshwater and ice) significantly reduced bacterial growth during extended storage (3–5 days) by minimizing fish temperature increases compared to fish boxes (high-density polyethylene/HDPE containers with layered fish and ice), widely used by the industry (55). To mitigate contamination during the transportation and storage, the study mandated using pre-cooled, intact containers with lids, maintaining near-0 °C water fully covering aquatic products, ensuring complete ice coverage over the water surface with timely replenishment, limiting storage to ≤5 days, and minimizing physical stress. Consistent temperature control via water circulation and cool environments is also essential for the vessels of storage and transportation. Besides the vessels, ice used in distribution or retail of aquatic product may sever as a source of microbial contamination, potentially transfer microbial cells to the products through cross-contamination (56). Once contaminated, the ice can act as both a reservoir and a vector, enabling microbial transfer during the storage, transportation, or retail display of aquatic products. Inadequate ice hygiene or repeated use of recycled ice further amplifies the risk. The study conducted by Atwill and Jeamsripong (57) reported that aquatic foods displayed on ice exhibited 1.7-fold higher prevalence of *Salmonella* contamination compared to those not stored on ice (*p* < 0.0001).

**Table 1:** Sources of microbial hazards along the aquatic supply chain.

Stage	Contamination source	Microbial hazards	Aquatic foods	References
Aquaculture	Water	Lactic acid bacteria, Aerobic mesophilic bacteria, *Enterobacteriaceae*, Total coliforms, and *Staphylococcus* spp.	Gilthead sea bream (*S. aurata*), Sea bass (*D. labrax*)	(45)
	Pond sediment, rearing water	*Shewanella Putrefaciens, Vibrio cholerae*	Fish (tilapia *Oreochromis niloticus*)	(86)
	Water	*Pseudomonas fluorescens*, *Aeromonas hydrophila*	Fish (*Thymallus thymallus L.*)	(87)
	Water, sediment	*Shewanella putrefaciens, Vibrio* spp.	Fish (*Oreochromis niloticus*)	(88)
	Water	*Salmonella,* SHV-12-producing *E. coli*	Trout	(89)
	Feed and water	*Streptoccocus agalactiae, Streptoccocus iniae, Staphyloccocus aureus*	Tilapia	(46)
Processing	Fishing vessel and processing plant	*Listeria monocytogenes*	Fish (mackerel, North Sea herring, North Sea- and Norwegian spring spawning herring, blue whiting, capelin)	(49)
	Fish processing plant (skinning machine, brine, guillotine, slicer conveyor roller, slicer control panel, slicer conveyor tape, rolls of fish bone remover, injector needle)	Heterotrophic plate count, *Enterobacteriaceae,* Total coliforms, *Listeria monocytogenes*	Fish (fish fillet, whole salmon, smoked fillet)	(90)
	Seafood processing plant (insulated vehicle, raw material receiving chute, fish basket, grading machine, grading table, soaking tank, weighing balance, weighing balance table, IQF table, block freezing conveyor belt, freezer pan, freezer, cooking boiler, depanning conveyor belt, wall tiles, cold store)	*Listeria monocytogenes*	/	(91)
	Tilapia-processing facilities	*Listeria monocytogenes*	Tilapia	(28)
	Salmon processing equipment	*Pseudomonas, Shewanella*	Salmon	(48)
Distribution/retail	Ice	Salmonella spp.	Blood cockle, Pacific white shrimp, oyster, Asian seabass	(57)
	Ice	*S. aureus, Salmonella, V. parahaemolyticus, L. monocytogenes*	Salmon	(56)

Additionally, microbial contamination during the retail stage of the aquatic food chain is influenced by personal hygiene of the handlers, packaging materials, and environmental conditions (58). For example, a study on edible bivalve shellfish found that the contamination rate of hepatitis A virus (HAV) was higher in samples from retail markets (6.1%) compared to those from aquaculture farms (1.8%) (59), highlighting the retail stage as a critical point for microbial hazards. In open-air wet markets, inadequate temperature control and minimal packaging easily lead to microbial growth. For instance, one study reported a 97% detection rate of *Escherichia coli* (*E. coli*) in live fish displayed at open-air wet markets, with an average concentration of 3.0 log CFU/g. And 58% of samples contained extended-spectrum beta-lactamase (ESBL)-producing *E. coli*, with an average concentration of 2.3 log CFU/g, and 28% tested positive for *Salmonella*. In contrast, modern supermarket, which employ cold chain systems, pre-packaging, and standardized protocols, exhibit lower contaminationlevels: 71% for *E. coli* (1.6 log CFU/g), 8% for ESBL-producing *E. coli* (1.6 log CFU/g), and 8% for *Salmonella* (60). These data indicated retail markets as critical high-risk points for microbial contamination, highlighting the need for improved hygiene practices to reduce microbial hazards along the aquatic food chain (61).

## 4 Traceability technologies in the aquatic supply chain

### 4.1 Conventional methods

Culture-dependent methods, as the gold standards, are widely used in the field of food safety, for the identification or counting of microorganisms along the aquatic supply chain (Supplementary Table 6) (62). By using selective media, specific bacteria (such as *Salmonella* spp., *Vibrio* spp., etc.) in aquatic products can be effectively isolated and identified (17). Generally, culture-dependent methods include the steps of sample preparation, enrichment, dilution, plating, enumeration, and isolation of single species colonies for further characterization (63). However, traditional culture methods often take a longer time to obtain results and do not provide real-time data.

### 4.2 Emerging technologies

With the advancement of technology, the emerging traceability technology provides a more efficient and accurate solution for the traceability of aquatic microorganisms, which can monitor and analyze the transmission path of microorganisms in real time.

#### 4.2.1 Molecular methods

Polymerase Chain Reaction (PCR) technology has been widely applied in the microbial traceability of aquatic products (Table 2) (64). Among its various forms, multiplex PCR enables the simultaneous amplification of multiple DNA fragments, thereby facilitating the detection of diverse microorganisms in aquaculture environments. Real-time PCR, when combined with high-resolution melting (HRM) curve analysis, supports high-throughput source tracking and can be integrated with production data to accurately identify contamination sources. This technique can also be integrated with production data to accurately pinpoint sources of contamination. To further enhance sensitivity and specificity, many studies have employed a two-round nested RT-PCR approach, which involves an initial RT-PCR followed by a second PCR using internal primers. For example, nested RT-PCR has been used to screen for hepatitis A virus (HAV) in mussels from Southern Italy, with the presence of infectious particles later confirmed through cell culture and RT-PCR (Supplementary Table 5) (65). In Galicia, a broad-spectrum nested RT-PCR based on Erker’s method and targeting the HEV ORF2 region was used to genotype HEV-positive samples (66). In addition, RT-booster-PCR has been developed to enhance detection sensitivity. This method involves a second amplification round using the same primers as those used in conventional RT-PCR. RT-booster-PCR has been successfully applied in the detection of norovirus, proving especially valuable as a complementary tool to RT-PCR when dealing with samples containing low viral loads or complex matrices (67). Due to its rapidity, sensitivity, and specificity, PCR technology holds significant promise for microorganism identification and contamination source tracing in the aquaculture industry.

**Table 2:** Comparisons of molecular detection methods for microorganisms along the aquatic supply chain.

Method	Sensitivity/Specificity	Applicable targets	Time & complexity	Cost	High-throughput suitability	References
PCR	High (both sensitivity and specificity)	Bacteria; viruses (RNA viruses require Reverse Transcription step)	Requires thermocycler; several hours; moderate technical demand	Low equipment cost; low reagent cost	Moderate (96-well plate)	(92)
qPCR (Real-time PCR)	High (comparable to conventional PCR)	Bacteria; viruses (requires Reverse Transcription for RNA)	Real-time monitoring; 1–2 h total; high technical requirement	High instrument cost; moderate consumables	High (multi-well plates; automated platforms)	(92)
Multiplex PCR	High (slightly below conventional PCR)	Multiple bacterial/viral targets	Similar runtime to conventional PCR; complex primer design	Similar equipment cost; higher primer cost	High (multiple targets per reaction)	(92)
LAMP	Very high	Bacteria; viruses (RT-LAMP for RNA)	Isothermal (60–65 °C); 1 h; simple setup	Low equipment cost; low reagent cost	Moderate (each reaction separate)	(93)
WGS	Extremely high (whole-genome resolution)	All microorganisms (bacteria, viruses, fungi, etc.)	Sample preparation + library construction + sequencing + bioinformatics; days; very complex	Very high instrument & analysis cost	Moderate (batch sequencing; large data)	(92)

Loop-mediated isothermal amplification (LAMP) represents a highly sensitive and specific approach for the detection of microorganisms in aquatic products (68). Through the utilization of a meticulously designed set of primers, LAMP enables the amplification of target microorganism nucleic acid sequences under a constant temperature condition. This allows for the accurate identification of multiple food - borne microorganisms present in fish, shellfish, and other aquatic product items. Compared with conventional techniques, LAMP stands out for its rapid turnaround, streamlined workflow and true on-site applicability, offering great potential to become an indispensable tool for microbial surveillance and traceability in the aquaculture industry. While LAMP is rapid, highly sensitive, and suitable for on-site detection, it requires complex primer design and may be prone to non-specific amplification. Future development could focus on improving multiplexing capability and integration with portable devices to enhance its applicability in diverse aquatic supply chain settings.

Whole genome sequencing (WGS) is increasingly employed as a culture-independent method for microbial traceability in aquatic products (69). Unlike traditional approaches such as 16S rRNA gene sequencing, which are limited in resolution due to targeting a single gene, WGS enables comprehensive analysis by examining the entire genome of microbial hazards. A typical WGS workflow integrates several bioinformatics tools: Fastp for read trimming and quality filtering; FastQC and MultiQC for quality assessment; Kraken2 for contamination detection; and SPAdes or Shovill for *de novo* genome assembly. QUAST is used to evaluate assembly quality, while Prokka performs genome annotation. Downstream comparative genomics analyses often involve tools such as MLST and cgMLST for typing, Snippy for single nucleotide polymorphism (SNP) detection, Roary for pan-genome analysis, and Mash for estimating genomic distances. These analyses facilitate the assessment of genetic relatedness among isolates for source attribution along the supply chain of aquatic products (70).

To infer evolutionary relationships, phylogenetic trees are constructed based on aligned core genome sequences. Maximum likelihood methods-implemented in tools like IQ-TREE, RAxML, and PhyML-provide robust phylogenetic inference under explicit evolutionary models. IQ-TREE, in particular, offers efficient and accurate tree estimation with integrated model selection and ultrafast bootstrap (UFBoot) support (71). For large datasets, approximate Maximum likelihood algorithms like FastTree or distance-based methods such as RapidNJ offer rapid generation of initial tree topologies. The reliability of phylogenetic inference critically depends on the appropriate choice of substitution models (e.g., GTR) and accounting for rate heterogeneity (e.g., using a gamma distribution). Source attribution and transmission pathway analyses combine genomic data with epidemiological metadata, including sampling time, geographic location, and exposure history (70). Isolates with high genomic similarity, typically clustering in the same monophyletic group and differing by no more than 20 core genome SNPs with strong bootstrap support, are considered to originate from a common source (70). In contrast, differences exceeding 100 SNPs generally indicated unrelated sources. For example, incorporating temporal and spatial metadata allows for the reconstruction of transmission chains, especially when early food or environmental isolates closely match clinical cases that emerge later (72). Based on WGS, some countries and regions have established public health networks for enhanced the surveillance of foodborne microorganism along the supply chain. Programs such as the FDA’s GenomeTrakr network highlight the power of WGS-based surveillance to rapidly identify contamination sources and support timely public health responses (72). Similarly, in the United States, PulseNet, led by the Centers for Disease Control and Prevention (CDC), is a molecular subtyping network for foodborne pathogens. Initially based on pulsed-field gel electrophoresis (PFGE), PulseNet has evolved to incorporate WGS, enabling standardized data sharing and real-time collaboration across laboratories to facilitate rapid outbreak detection and source tracing (73). In China, a national foodborne disease molecular tracing network (TraNet) has been established to address foodborne diseases (74). The establishment of this network provides strong technical support for identifying the causes and accurately tracing the source of foodborne disease outbreaks across regions and even internationally. However, WGS, while offering unmatched resolution for microbial source attribution, remains limited in routine aquatic product monitoring by high costs, data processing requirements, and the need for specialized bioinformatics expertise. Advances in portable sequencing platforms and automated analysis pipelines may broaden its accessibility and facilitate near real-time source tracking.

#### 4.2.2 Biomarkers for aquatic product microbiology traceability

Microbial DNA profiling serves as a direct biological fingerprint for tracing the origin of microbiological communities on aquatic products. By characterizing the unique composition of a microbial community, a field known as microbial biogeography, this method can link a sample to a specific geographic region based on its distinctive microbial signature (75). Concurrently, stable isotope analysis of the host organism acts as a robust geochemical tracer, determining the geographic origin of the aquatic product itself (76). By revealing where the host fish lived and foraged, this analysis provides a powerful environmental context, or a geographical proxy, for the microbial community it harbors.

**Figure 3:**
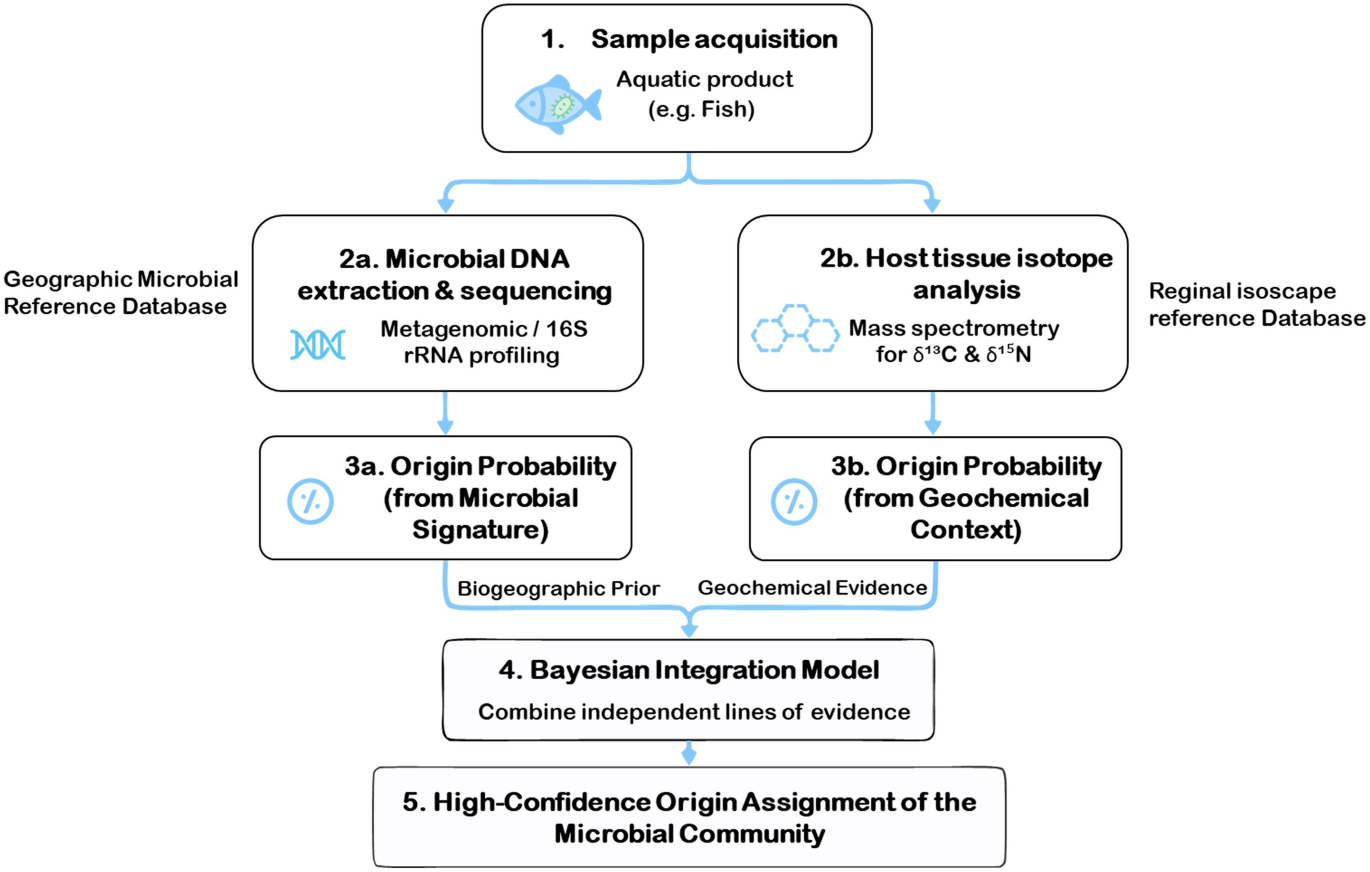
A framework for tracing the origin of microbial communities in the aquatic supply chain (75, 76).

The advancement presented herein lies in the synergistic combination of microbial DNA profiling and host isotopic geolocation, which collectively enhance the precision of origin assignment within a unified analytical framework. By employing an advanced analytical framework, such as a Bayesian model, the probabilistic origin assignment from the microbial DNA (the direct biological evidence) can be powerfully refined using the geographical origin data from the host’s isotopes (the environmental context) (Figure 3). This dual-layered approach transforms two separate analyses into a single, high-confidence verification system. The result is a multidimensional and exceptionally robust traceability framework, capable of pinpointing the geographic source of microbial communities on aquatic products with unprecedented precision.

#### 4.2.3 Applications of AI in microbial source tracking of aquatic products

In recent years, the integration of artificial intelligence (AI) and big data technologies into microbial source tracking has shown immense promise, particularly for ensuring the safety of aquatic products. AI methods, especially machine learning (ML), can simulate human intelligence to analyze large-scale genomic and microbiome datasets, thereby significantly enhancing the speed and accuracy of contamination source identification.

The primary application of AI in the source tracking of aquatic products involves direct tracing based on the inherent genetic information or community structures of microorganisms, which act as unique “identity fingerprints.” When tracing specific microbial contamination events along the supply chain, WGS provides forensic-level precision. For instance, U.S. agencies including the Centers for Disease Control and Prevention (CDC), the Food and Drug Administration (FDA), and the Department of Agriculture (USDA) have co-developed machine learning models trained on WGS data to accurately estimate the sources of *Salmonella* infections, achieving remarkable accuracy (77). The core workflow of this approach involves creating standardized whole-genome multilocus sequence typing (wgMLST) allelic profiles from bacterial WGS data to serve as ‘genomic fingerprints’. A Random Forest machine learning model is then trained on a reference database of these fingerprints from known origins. Finally, the fingerprint of an unknown isolate is fed into this trained model to generate a probability report pinpointing its most likely source.

Beyond tracking a single microorganism, AI can leverage an entire microbial community as a “natural barcode” to verify the geographic origin of aquatic products. This capability is crucial for ensuring supply chain integrity and safety, as it helps confirm that a product is genuinely from its stated source. In one study, researchers combined 16S rRNA gene metabarcoding with supervised machine learning algorithms to successfully trace the geographic origin of aquatic products (78). This approach begins by sequencing the V3-V4 hypervariable region of the bacterial 16S rRNA gene from two clam tissues, the gills and digestive glands, to construct Amplicon Sequence Variant (ASV) profiles that represent the microbial community composition. As a key step, these ASV profiles are converted into binary “presence/absence” matrices to serve as input features for machine learning. Specific models were trained for different traceability objectives: a Bagging-enhanced multinomial logistic regression for provenance tracking (a multi-class task) and a Random Forest to distinguish between legal and illegal harvesting (a binary-class task). To ensure reliability, all models were trained using sample data from the second year and validated on a temporally independent dataset from the first year. Finally, a “consensus model” was created by fusing the prediction probabilities from the separate models for gills and digestive glands to further improve the final traceability accuracy.

As microbiome datasets grow in scale and dimensionality, the performance of traditional machine learning methods can face bottlenecks. To address this challenge, researchers have introduced advanced deep learning frameworks like the Ontology-aware Neural Network for Microbiome Source Tracking (ONN4MST) (79). The method’s workflow begins by preparing the input data: microbial community abundance profiles paired with a predefined hierarchical map of sample environments, known as a biome ontology. A crucial subsequent step employs a random forest algorithm for intelligent feature selection, identifying a core set of approximately 1,462 key indicators from the vast number of microbial species. This streamlined data is then used to train the central neural network, which learns to associate specific microbial ‘fingerprints’ with their precise, multi-layered location within the biome ontology. Once trained, the model can make predictions with just a single forward pass. This efficient architecture allows ONN4MST to achieve millisecond-level response times (about 20 s for 100 samples) on datasets with nearly one million samples, all while maintaining a low memory footprint (around 22 GB). Critically, compared to traditional tools like FEAST and SourceTracker, its accuracy improves from 0.89 to 0.98 in benchmark tests against tools like FEAST and SourceTracker, representing a comprehensive optimization of speed, memory, and precision.

**Figure 4:**
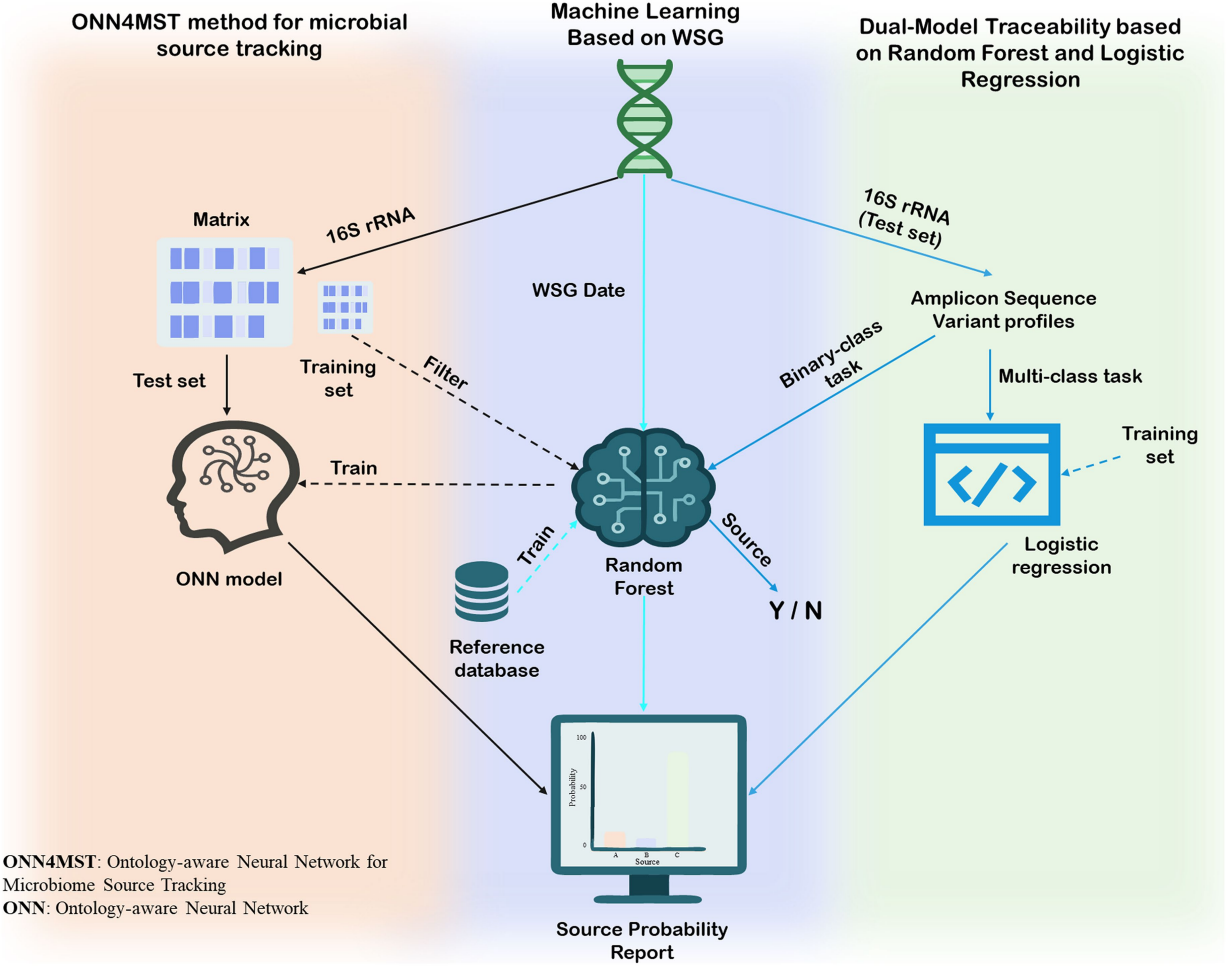
Application of three artificial intelligence methods in microbial source tracking in the aquatic supply chain.

In summary, artificial intelligence and big data are fundamentally reshaping the landscape of microbial source tracking for aquatic products. From high-precision, genome-based pathogen tracing and microbial community-based origin verification to the development of advanced deep learning frameworks for large-scale data analysis, machine learning provides scalable, high-throughput solutions for accurately identifying and verifying microbial sources in aquatic products. As computational algorithms and microbiome databases continue to evolve, AI is poised to play an increasingly vital role in safeguarding the microbial safety and traceability of aquatic food products. AI enables high-accuracy microbial source tracking by integrating complex multi-dimensional datasets, but its performance depends heavily on the availability of large, diverse, and high-quality training data. Combining AI with complementary approaches such as WGS or stable isotope analysis could improve predictive robustness and practical adoption in regulatory frameworks (see Figure 4).

## 5 Challenges in aquatic product traceability

### 5.1 Technical and data barriers

One of the primary technical hurdles in the microbial traceability of aquatic products is the lack of uniformity in technology adoption among different countries and regions (80). Developed nations generally have the requisite infrastructure, technological capabilities, and regulatory frameworks to implement efficient traceability systems. Conversely, numerous developing countries, which are significant sources of aquatic products, frequently lack the essential technology, equipment, and skilled workforce to establish comparable systems.

Furthermore, the internal systems utilized by enterprises, such as Enterprise Resource Planning (ERP) and Laboratory Information Management Systems (LIMS), often diverge from government regulatory platforms in terms of data formats and transmission protocols. In practice, mechanisms for verifying data authenticity and preventing tampering prior to uploading, such as blockchain technology, are under - exploited. This makes it arduous to guarantee the auditability of the traceability chain. All these factors have a detrimental impact on the microbial traceability of aquatic products.

### 5.2 Policy and standardization gaps

Currently, there is no dedicated international standard specifically addressing the traceability of microbial hazards in aquatic products. Most countries base their systems on the general principles of the Codex Alimentarius and their respective national frameworks such as Regulation (EC) No 178/2002 in the European Union and China’s Food Safety Law. However, the depth and specificity of these standards vary widely (81, 82). Existing traceability systems typically emphasize batch origin and distribution pathways, but often lack harmonized provisions on microbial threshold limits, quantitative risk assessment methodologies, and laboratory capability verification mechanisms.

Furthermore, significant regional disparities exist in the enforcement and implementation of these standards. Regulatory bodies in developed countries, such as the U.S. FDA and Japan’s Ministry of Health, Labour and Welfare, have established mature practices regarding regulatory oversight, enterprise audits, sample testing, and legal enforcement. In contrast, many developing countries face limitations in regulatory infrastructure and enforcement capacity, which undermine their ability to establish effective deterrents against microbial hazards in aquatic supply chains.

This disparity underscores the urgent need for globally harmonized traceability standards and capacity-building efforts to ensure consistent food safety governance across regions.

### 5.3 Challenges of traceability in complex supply chains

The aquatic product supply chain is intricate and globally dispersed. Traceability can be easily disrupted when responsibilities are fragmented among numerous stakeholders. Aquaculture and capture fisheries involve harvesters, processors, distributors, and retailers, who may be located in different countries and subject to different authorities (e.g., fisheries management agencies and food safety regulators). Insufficient coordination often gives rise to gaps. A report on Southeast Asian fisheries noted that government oversight is plagued by fragmented responsibilities across agencies and regional authorities, and enforcement loopholes impede effective monitoring (83).

Likewise, the existence of multiple private and public traceability systems, such as eco-labels, catch certificates, and import controls, can lead to overlaps without integration, resulting in confusion regarding responsibilities. These disconnects delay recall actions. Outbreak investigations may require piecing together invoices, Hazard Analysis and Critical Control Points (HACCP) records, and catch reports from various sources (84). Even when traceability data is available, complex processing procedures (e.g., the mixing of frozen fish batches) make it difficult to correlate microorganism test results with final products. In essence, multi-layered supply chains, cross-border trade, and siloed institutions reduce transparency.

## 6 Future perspectives

The development of aquatic microorganism traceability will greatly benefit from innovations in microbial detection methods. Highly accurate and rapid detection technologies will help identify contamination promptly, enabling timely responses and corrective measures. Developing mature on-site detection technologies, especially those that do not destroy the samples, can combine both cost-effectiveness and practical monitoring results, thus lowering the detection threshold and promoting widespread adoption. Additionally, continuous monitoring of environmental parameters such as water temperature, pH, dissolved oxygen, and turbidity in aquaculture systems can also serve as early warning indicators for microbial risk, further enhancing prevention and control capabilities.

However, building a full-chain traceability system for microorganisms faces significant challenges in handling vast amounts of data. Due to difficulties in data sharing and lack of interoperability between different stages of the supply chain, more intelligent tools, such as artificial intelligence, big data analysis, and blockchain technology, are urgently needed to improve data integration and risk identification. Therefore, developing and integrating intelligent systems to support aquatic microorganism traceability is of paramount importance.

The development of such technologies also requires guidance and support from policies and regulations. A review concluded that although many countries have implemented HACCP systems and corresponding inspection regulations, coordination between regulations remains necessary in managing hazards within increasingly globalized supply chains (85). Governments can promote the establishment of mandatory microorganism genome data upload and sharing mechanisms through legislation, building a global foodborne microorganism database, which is crucial for responding swiftly to international food safety incidents.

## 7 Conclusion

Aquatic products play a vital role in global food security, especially in regions abundant in marine resources. However, with the expansion of international trade, ensuring the microbial safety of these products has become an increasingly complex challenge. Foodborne microorganisms associated with aquatic products continue to pose serious public health risks and economic burdens.

Traceability technologies have emerged as essential tools for addressing these challenges. By enabling real-time monitoring and precise tracking of each stage in the aquatic product supply chain, these systems significantly enhance the ability to prevent, detect, and respond to microbial contamination. The integration of advanced technologies such as Internet of Things (IoT) sensors, genome sequencing, and big data analytics further strengthens the effectiveness and responsiveness of traceability frameworks.

Despite these advancements, significant obstacles remain. The uneven access to technological infrastructure, regulatory fragmentation across borders, and limited consumer awareness hinder the full potential of traceability systems. To move forward, efforts should focus on developing cost-effective and scalable traceability solutions, harmonizing international standards, enhancing policy frameworks, and promoting public engagement.

Prospectively, the integration of advanced technologies, including synthetic biology, artificial intelligence, and real-time data platforms, holds great promise for developing more robust and intelligent traceability systems. These innovations will contribute to achieving a safer, more transparent, and sustainable global aquatic product supply chain.
