# Supplementary material for: Tracing microbial hazards in the aquatic supply chain: challenges, technologies, and future directions
Source: Front Nutr. 2025 Oct 1;12:1673037. doi: 10.3389/fnut.2025.1673037 (PMC12520881; doi:10.3389/fnut.2025.1673037)
Supplement: Supplementary file 1 [file Table_1.docx]

Supplementary file

Supplementary Table 1 Detection and prevalence of *Vibrio* spp. in aquatic foods, 2020-2025

| Aquatic food | Prevalence (%) | Detection technique | Country/Region | Sample site | References |
| --- | --- | --- | --- | --- | --- |
| Tasmanian bivalve shellfish | *Vibrio parahaemolyticus* (8-100 %) | Most Probable Number (MPN), qPCR (*ldh*, *tdh*, *trh*, *vvh* genes) | Australia | Coasts | [1] |
| Tuna (n = 108), pelagic fish (n = 78), cephalopod (n = 37), sardine (n = 14), demersal  fish (n = 53) | *Vibrio parahaemolyticus* (95%) | PCR (*vp*) | Bali province | Traditional fish markets | [2] |
| Oreochromis niloticus: moribund fish (n = 120), apparently healthy (n = 60) | *Vibrio parahaemolyticus* (8.9%) | PCR (*toxR*, *tlh, tdh*,*trh*) | Egypt | Lake Manzala | [3] |
| Black tiger shrimps, white-legged shrimp, red shrimps, blue mussels, oysters, scallop meat | *V. parahaemolyticus* (58%), *V. alginolyticus* (42%), *V. cholerae* non-O1/non-O139 (25%), *V. vulnificus* (4%) | Multiplex PCR  (*ctxA*, *tdh*, *trh* genes) | Germany | Supermarkets and fish markets | [4] |
| Fish, shrimp, shellfish, crab, cephalopod | *V. Parahaemolyticus* (8.2%), *V. campbellii* (2.5%) | Culture-dependent method (TCBS) | China | Retail market | [5] |
| Crayfish | *V. Parahaemolyticus* (65%) | Culture-dependent method (TCBS and CHROMagar™) | China | Crayfish farms, wholesale, retail aquatic products market | [6] |
| Villorita cyprinoides (n = 24) | *V. Parahaemolyticus* (55.6%) | Multiplex PCR  (*tlh, tdh, trh* genes), simplex PCR (*toxR* gene) | India | Coast | [7] |
| Shrimp, shellfish, squid | *V. Parahaemolyticus* (21.7%) | Culture-dependent method (TCBS) and multiplex PCR (*ldh*, *tdh*, *trh* genes) | Thailand | Wet markets and supermarkets | [8] |
| Frozen Bivalve Molluscs | Viable but Nonculturable *Vibrio parahaemolyticus* (11.7%) | Culture-dependent method (TCBS), qPCR, PMA-qPCR (*tlh* gene) | Italy | Mass market | [9] |
| Fish, shrimp, shellfish, freshwater | *Vibrio parahaemolyticus* (47.0%) | Culture-dependent method (TCBS) and PCR (*tlh* gene) | China | Retail market | [10] |
| Mussels (n = 20), veined rapa whelks (n = 20), oysters (n = 20), Bluefish (n = 10), horse mackerel (n = 20), gilthead seabream (n = 20), sea bass (n = 20), atlantic salmon (n = 20), whiteleg shrimp (n = 10), argentine shortfin squid (n = 20) | *Vibrio parahaemolyticus* (24%) | PCR (*toxR*, *tdh*,*trh* genes) | Bulgaria | Retail shops | [11] |
| Shrimp and crab (n = 89), fish (n = 276), shellfish  (n = 189) and Cephalopods  (n = 101) | *V. vulnificus*  (9.01%) | Novel visual loop-mediated isothermal amplification (LAMP) assay (*gyrB* target) | China | Café, supermarket, farmers’ market and online store | [12] |
| Shrimp: *P. monodon* (n = 150), *P. vannamei* (n = 60) | *Vibrio* spp.  (15%) | PCR (*pyrH* gene) | Malaysia | Farms | [13] |
| Fish (n = 200), shellfish (n = 200) | *Vibrio parahaemolyticus* (fish, 7.5%; shellfish, 13.0%) | PCR (16S rRNA genes) | United Arab Emirates | Markets | [14] |
| Clam  (n=30) | *Vibrio parahaemolyticus* (33.3%) | LAMP-LFD assay (*bla_CARB-17_* gene) | China | Retail market | [15] |
| Fish  (n = 80) | *Vibrio parahaemolyticus* (65.37%) | Multiplex PCR (*tlh*, *tdh*, *trh* genes) | Bangladesh | Retail market | [16] |
| Fish  (n = 33) and mussels  (n = 64) | *V**ibrio parahaemolyticus* (22.7%) | Culture-dependent method (TCBS) and PCR (*toxR* gene) | Bulgaria | Regional marketplaces | [17] |
| Fish  (n = 300) | *Vibrio parahaemolyticus* (14%), *Vibrio alginolyticus* (16.7%) | Culture-dependent method (TCBS) and multiplex PCR (*toxR*, *vvhA*, *vP, vA*, *tdh*, *trh*, *tlh* genes) | Egypt | Wet markets and supermarkets | [18] |
| Red seabreams (n = 8), Korean rockfish (n =29), whiteleg shrimp (n = 11) | *Vibrio parahaemolyticus* (34.7%) | Culture-dependent method (TCBS), oxidase production, fermentation activity and VITEK system | Korea | Aquaculture production areas | [19] |
| Freshwater fish (n = 250) | *Vibrio parahaemolyticus* (19.2%) | Multiplex PCR (*tlh*, *tdh*, *trh* genes) | Dakahlia governorate | Farm | [20] |
| Pacific oysters (n = 693) | *Vibrio parahaemolyticus* (85%) | PCR (*toxR*, *vvha* genes) | New Zealand | Island | [21] |

Supplementary Table 2 Detection and prevalence of *Salmonella* spp. in aquatic foods, 2020-2025

| Aquatic food | Prevalence | Detection technique | Country/Region | Sample site | References |
| --- | --- | --- | --- | --- | --- |
| Aquatic product (n=240) | *Salmonella* spp*.* (4.58%) | Culture-dependent method, PCR assay (*invA* gene), WGS | China | Local agriculture market | [22] |
| Shrimp, crab, clam, scallops, grass carp, crucian carp, perch, and squid (n=150) | *Salmonella* spp*.* (2.7%) | Culture-dependent method (US-FDA Bacteriological Analytical Manual (BAM)-*Salmonella*),  chip-based digital LAMP (cdLAMP) | China | Seafood market | [23] |
| Seafood (n=50) | *Salmonella* spp*.* (64%) | Culture-dependent method (ISO 6579-1:2017) | Cambodia | Local market | [24] |
| Tilapia, shrimp (n=324) | *Salmonella* spp*.* (11%) | Culture-dependent method, PCR, WGS | Bangladesh | Wet market | [25] |
| Mussels (n=2115), oysters (n=250) and clams (n=65) | *Salmonella* spp*.* (0.6%) | Culture-dependent method (ISO 66579-1) | Italy | Wholesale, retail market | [26] |
| Freshwater fish, saltwater fish, and shrimp (n=300) | *Salmonella* spp*.* (5.3%) | Culture-dependent method (GB 4789.4-2010);  MPN method (USDA/FSIS. Most probable number procedure and tables) | China | Retail market | [27] |

Supplementary Table 3 Detection and prevalence of *Listeria monocytogenes* in aquatic foods, 2020-2025

| Aquatic food | Prevalence | Detection technique | Country/Region | Sample site | References |
| --- | --- | --- | --- | --- | --- |
| Fishe, mussel, shrimp, squid, crab and octopu  (n = 625) | *Listeria monocytogenes*  (5.92%) | Culture-dependent method; PCR assay (*inlA* gene) | Türkiye | Market | [28] |
| Dead raw fish(n = 750) | *Listeria monocytogenes*  (3.21%) | Culture-dependent method (PN-EN ISO 11290-1:2017-07); PCR assay (*prs* gene) | Poland | Standing freshwater tanks, flowing freshwater and a saltwater reservoir | [29] |
| Fish  (n = 165) | *Listeria monocytogenes*  (11.5%) | Culture-dependent method, multiplex PCR (*prs, lmo0737, lmo1118, ORF2819, and ORF2110* genes) | Greece | Fishmongers | [30] |
| Striped  red mullet, whiting (n=500) | *Listeria monocytogenes* (0.2%) | Culture-dependent method (EN ISO 11290-1), PCR | Turkey | Commercial fish collection area | [31] |
| Cold smoked salmon  (n = 160) | *Listeria monocytogenes* (24.05%) | PCR assay (*Iap* gene) | Lithuania | Retail market | [32] |

Supplementary Table 4. Detection and prevalence of *Pseudomonas* spp. in aquatic foods, 2020-2025

| Aquatic food | Prevalence | Detection technique | Country/Region | Sample site | References |
| --- | --- | --- | --- | --- | --- |
| Nile tilapia, Golden grey mullet, Mediterranean mackere, Striped red mullet (n=276) | *Pseudomonas aeruginosa* (57.9%) | Culture-dependent method (ISO 3720) | Egypt | Fish market | [33] |
| Fish fillet  (n = 75) | *Pseudomonas* spp.  (9.9%) | Culture-dependent method (UNI EN ISO 13720:2010), oxidase test, Kligler Iron Agar infixion test | Italy | Fish industry | [34] |
| Lobster  (n=92) | *Pseudomonas aeruginosa* (8.7%) | Culture-dependent method | Iran | Wholesale seafood market | [35] |
| Fish  (n = 252) | *P. Aeroginosa* (14.43%)*, P. fluorescen* (8.75%)*, and P.*  *anguilliseptica* (4.75%) | Culture-dependent method | Egypt | Fish farm, | [36] |
| Fish  (n = 285) | *Pseudomonas aeruginosa* (31.57%) | Culture-dependent method, PCR assay (16S rRNA gene) | Egypt | Private freshwater farm | [37] |

Supplementary Table 5 Detection and prevalence of norovirus in aquatic foods, 2020-2025

| Aquatic food | Prevalence | Detection technique | Country/Region | Sample site | References |
| --- | --- | --- | --- | --- | --- |
| Oyster and mussel (n=168) | Norovirus  (32.7%) | qRT-PCR | Italy | Farming area | [38] |
| Mussels and oysters (n=134) | Norovirus  (40.3%) | qRT-PCR | Brazil | Producer and local market | [39] |
| Shellfish (n=246) | Norovirus  (16.7%) | qRT-PCR | Poland | Seafood wholesale | [40] |
| Oyster (n=10) | Norovirus  (60%) | ELSA | Mauritius | Aquaculture farm | [41] |
| Oyster (n=337) | Norovirus  (2018-2019: 94.3%; 2019-2020: 96.6%; 2020-2021: 63.2%)  Varied among various years | qRT-PCR | Ireland | Oyster production area | [42] |
| Mollusks (n=2266) | Norovirus  (8.1%) | qRT-PCR | Italy | Aquaculture plant | [43] |
| Mollusks （n=162) | Norovirus  (8.02%) | qRT-PCR | Italy | Harvesting, depuration, sipping, restaurant, market and retail store | [44] |
| Oyster and mussel (n=380) | Norovirus  (0%) | qRT-PCR | Brazil | Harvesting area, beach vendor | [45] |
| Oyster and mussel (n=77) | Norovirus  (41.5%) | qRT-PCR | Brazil | Shellfish growing area | [46] |
| Oysters, clams, shrimps and finfish (n=104) | Norovirus GII (41.34%) | RT-PCR, southern hybridization | India | Retail marker and loading center | [47] |
| Pacific oysters and white hard clams (n=121) | Norovirus GI (50.4%), Norovirus GII (79.3%) | qRT-PCR | Vietnam | Retail, fish market, supermarket | [48] |

Supplementary Table 6 Detection and prevalence of hepatitis viruses in the aquatic foods, 2020-2025

| Aquatic food | Prevalence | Detection technique | Country/Region | Sample site | References |
| --- | --- | --- | --- | --- | --- |
| Oyster and mussel (n=168) | HAV  (0%) | qRT-PCR | Italy | Shellfish farming area | [38] |
| Mollusks (n=2266) | HAV  (0.26%) | qRT-PCR | Italy | Aquaculture plant | [43] |
| Mollusks （n=162) | HAV (0.62%), HEV (0.62%) | qRT-PCR | Italy | Harvesting, depuration, sipping, restaurant, market and retail store | [44] |
| Fish and shrimp (n=320) | HAV (0.93%) | qRT-PCR | Iran | Local harbor and retail center | [49] |
| Clam  (n=14) | HAV  (64.3%) | RT-PCR | Africa | River | [50] |
| Pacific oysters and white hard clams (n=121) | HAV (1.7%), HEV (11.6%) | qRT-PCR | Vietnam | Retail, fish market, supermarket | [48] |

Supplementary Table 7 Standard detection methods for common microbial hazards in aquatic foods

| Microbial hazards | Detection methods | Organization | References |
| --- | --- | --- | --- |
| *Salmonella* spp. | Culture dependent method (NIHSJ-01-ST4) | National Institute of Health Science (NIHS) | [51] |
|  | Culture dependent method (AOAC O.M. 2001.07) | Association of Official Analytical Collaboration (AOAC) International | [52] |
|  | Culture dependent method (AS 5013.10:2022) | Standards Australia | [53] |
|  | Culture dependent method (FDA BAM Ch.5) | Food and Drug Administration (FDA) | [54] |
|  | Culture dependent method (ISO 6579‑1:2017) | International Organization for Standardization (ISO) | [55] |
|  | Culture dependent method (GB 4789.4-2024) | National Health Commission of the People's Republic of China & State Administration for Market Regulation (NHC & SAMR) | [56] |
| *Vibrio* spp. | PCR (Microbiological Methods & Bacteriological Analytical Manual (BMA)) | FDA | [54] |
|  | Culture-dependent method (ISO 21872-1:2017) | ISO | [57] |
|  | Culture-dependent method (GB 4789.7-2013) | National Health and Family Planning Commission of the People's Republic of China (NHFPC) | [58] |
| *Listeria monocytogenes* | Culture-dependent method (ISO 11290-1:2017; GB 4789.30-2016) | China Food and Drug Administration (CFDA) & NHFPC | [59] |
|  | Culture-dependent method (BMA) | FDA | [54] |
|  | Culture-dependent method (ISO 11290-1:2017) | ISO | [60] |
| Norovirus | RT-PCR (SE-751 26) | European Union Reference Laboratory for Foodborne Viruses (EU-RL FV) | [61] |
|  | RT-PCR (ISO 15216-2:2019) | ISO | [62] |
|  | RT-PCR (GB4789.42-2016) | NHC & SAMR | [56] |
| Hepatitis A virus | RT-PCR (SE-751 26) | EU-RL FV | [61] |
|  | RT-qPCR (ISO 15216-2:2019) | ISO | [62] |

**References**

[1] Torok, V. A., Mahbub, K. R., Grey, P. A., Fletcher, G. C., & Turnbull, A. R. (2025). Survey of foodborne pathogenic *Vibrio* species in commercial Tasmanian bivalve shellfish and predictors of risk at harvest [Journal Article]. *International Journal of Food Microbiology*, 430, 111033. https://doi.org/10.1016/j.ijfoodmicro.2024.111033

[2] Sudaryatma, P. E., Wiradana, P. A., Razaq, I., Sunarsih, N. L., Jatmiko, A., Permatasari, A. A. A. P., Sari, N. K. Y., Widhiantara, I. G., Sandhika, I. M. G. S., & Rosiana, I. W. (2025). Prevalence of bacterial contamination on seafoods products collected from traditional fish market in Bali Province during 2023: Prevalensi kontaminasi bakteri pada produk hasil ikan laut yang dikumpulkan dari pasar ikan tradisional di Provinsi Bali selama tahun 2023. *Jurnal Pengolahan Hasil Perikanan Indonesia*, 28(3), 297-309. https://doi.org/10.17844/jphpi.v28i3.61627

[3] Algammal, A. M., Mabrok, M., Alfifi, K. J., Alghamdi, S., Almanzalawi, E. A., Alqahtani, T. M., Elsayed, M. E., Elghayaty, H., El Kattawy, Z. M., & El-Tarabili, R. M. (2024). The prevalence, antimicrobial susceptibility, virulence, and antimicrobial resistance genes of multidrug-resistant *Vibrio parahaemolyticus* recovered from Oreochromis niloticus. *Aquaculture International*, 32(7), 9499-9517. https://doi.org/10.1007/s10499-024-01625-x

[4] Zeidler, C., Szott, V., Alter, T., Huehn-Lindenbein, S., & Fleischmann, S. (2024). Prevalence of *Vibrio* spp. in Seafood from German Supermarkets and Fish Markets *Foods*, 13(24), 3987. https://doi.org/10.3390/foods13243987

[5] Huang, Q., Zhang, Y., Zhang, M., Li, X., Wang, Q., Ji, X., Chen, R., Luo, X., Ji, S., & Lu, R. (2024). Assessment of *Vibrionaceae* prevalence in seafood from Qidong market and analysis of *Vibrio parahaemolyticus* strains. *Plos One*, 19(8), e309304. https://doi.org/10.1371/journal.pone.0309304

[6] Wu, K., Zou, D., Long, Y., Xue, L., Shuai, S., Tian, F., Li, M., Fan, G., Zheng, Y., Sun, X., Wang, W., Wang, L., Ni, X., Zhang, X., Fan, Y., & Li, H. (2024). Contamination of *Vibrio parahaemolyticus* in crayfish for sale. *Frontiers in Microbiology*, 15, 2024. https://doi.org/10.3389/fmicb.2024.1388658

[7] Antony, A. C., Silvester, R., Aneesa, P. A., P. V., V., Selvam A, D. G., Salim, V., Paul, M. K., & Abdulla, M. H. (2024). Occurrence, virulence, and AMR profile of *Vibrio parahaemolyticus* isolated from shellfish growing areas located along the south-west coast of India. *Journal of Water and Health*, 22(9), 1594-1605. https://doi.org/10.2166/wh.2024.338

[8] Siriphap, A., Prapasawat, W., Borthong, J., Tanomsridachchai, W., Muangnapoh, C., Suthienkul, O., & Chonsin, K. (2023). Prevalence, virulence characteristics, and antimicrobial resistance of *Vibrio parahaemolyticus* isolates from raw seafood in a province in Northern Thailand. *Fems Microbiology Letters*, 371, fnad134. https://doi.org/10.1093/femsle/fnad134

[9] Di Salvo, E., Panebianco, F., Panebianco, A., & Ziino, G. (2023). Quantitative Detection of Viable but Nonculturable *Vibrio parahaemolyticus* in Frozen Bivalve Molluscs. *Foods*, 12(12), 2373.

[10] Li, M., Xu, H., Tian, Y., Zhang, Y., Jiao, X., & Gu, D. (2023). Comparative genomic analysis reveals the potential transmission of *Vibrio* *parahaemolyticus* from freshwater food to humans. *Food Microbiology*, 113, 104277. https://doi.org/10.1016/j.fm.2023.104277

[11] Stratev, D., Fasulkova, R., & Krumova-Valcheva, G. (2023). Incidence, virulence genes and antimicrobial resistance of *Vibrio parahaemolyticus* isolated from seafood. *Microbial Pathogenesis*, 177, 106050. https://doi.org/https://doi.org/10.1016/j.micpath.2023.106050

[12] Tian, Z., Yang, L., Qi, X., Zheng, Q., Shang, D., & Cao, J. (2022). Visual LAMP method for the detection of *Vibrio vulnificus* in aquatic products and environmental water. *Bmc Microbiology*, 22(1), 256. https://doi.org/10.1186/s12866-022-02656-1

[13] Haifa-Haryani, W. O., Amatul-Samahah, M. A., Azzam-Sayuti, M., Chin, Y. K., Zamri-Saad, M., Natrah, I., Amal, M. N., Satyantini, W. H., & Ina-Salwany, M. Y. (2022). Prevalence, Antibiotics Resistance and Plasmid Profiling of *Vibrio* spp. Isolated from Cultured Shrimp in Peninsular Malaysia *Microorganisms*, 10(9), 1851. https://doi.org/10.3390/microorganisms10091851

[14] Abdalla, T., Al-Rumaithi, H., Osaili, T. M., Hasan, F., Obaid, R. S., Abushelaibi, A., & Ayyash, M. M. (2022). Prevalence, Antibiotic-Resistance, and Growth Profile of *Vibrio* spp. Isolated From Fish and Shellfish in Subtropical-Arid Area. *Frontiers in Microbiology*, 6(13), 861547. https://10.3389/fmicb.2022.861547.

[15] Hu, Y. Q., Huang, X. H., Guo, L. Q., Shen, Z. C., Lv, L. X., Li, F. X., Zhou, Z. H., & Zhang, D. F. (2021). Rapid and Visual Detection of *Vibrio parahaemolyticus* in Aquatic Foods Using bla(CARB-17) Gene-Based Loop-Mediated Isothermal Amplification with Lateral Flow Dipstick (LAMP-LFD). *Journal of Microbiology and Biotechnology*, 31(12), 1672-1683. https://doi.org/10.4014/jmb.2107.07022

[16] Ali, S., Hossain, M., Azad, A. B., Siddique, A. B., Moniruzzaman, M., Ahmed, M. A., Amin, M. B., Islam, M. S., Rahman, M. M., Mondal, D., & Mahmud, Z. H. (2021). Diversity of *Vibrio parahaemolyticus* in marine fishes of Bangladesh. *Journal of Applied Microbiology*, 131(5), 2539-2551. https://doi.org/10.1111/jam.15093

[17] Stratev, D., Stoyanchev, T., & Bangieva, D. (2021). Occurrence of *Vibrio parahaemolyticus* and Staphylococcus aureus in seafood. *Italian Journal of Food Safety*, 10(4), 10027. https://doi.org/10.4081/ijfs.2021.10027

[18] Sadat, A., El Sherbiny, H., Zakaria, A., Ramadan, H., & Awad, A. (2021). Prevalence, antibiogram and virulence characterization of *Vibrio* isolates from fish and shellfish in Egypt: a possible zoonotic hazard to humans. *Journal of Applied Microbiology*, 131(1), 485-498. https://doi.org/10.1111/jam.14929

[19] Mok, J. S., Cho, S. R., Park, Y. J., Jo, M. R., Ha, K. S., Kim, P. H., & Kim, M. J. (2021). Distribution and antimicrobial resistance of *Vibrio* *parahaemolyticus* isolated from fish and shrimp aquaculture farms along the Korean coast. *Marine Pollution Bulletin*, 171, 112785. https://doi.org/10.1016/j.marpolbul.2021.112785

[20] Zaher, H. A., Nofal, M. I., Hendam, B. M., Elshaer, M. M., Alothaim, A. S., & Eraqi, M. M. (2021). Prevalence and Antibiogram of Vibrio parahaemolyticus and Aeromonas hydrophila in the Flesh of Nile Tilapia, with Special Reference to Their Virulence Genes Detected Using Multiplex PCR Technique. Antibiotics, 10(6), 654. https://doi.org/10.3390/antibiotics10060654

[21] King, N. J., Pirikahu, S., Fletcher, G. C., Pattis, I., Roughan, B., & And Perchec Merien, A. (2021). Correlations between environmental conditions and *Vibrio parahaemolyticus* or *Vibrio vulnificus* in Pacific oysters from New Zealand coastal waters. *New Zealand Journal of Marine and Freshwater Research*, 55(3), 393-410. https://doi.org/10.1080/00288330.2020.1796718

[22] Jiang, X., Siddique, A., Chen, L., Zhu, L., Zhou, H., Na, L., Jia, C., Li, Y., & Yue, M. (2025). Genomic and resistome analysis of *Salmonella* enterica isolates from retail markets in Yichun city, China. *One Health*, 20, 100967. https://doi.org/https://doi.org/10.1016/j.onehlt.2025.100967

[23] Chen, A., Ma, B., Zhang, Y., Huang, W., Xiao, Y., Li, J., Tang, Q., & Zhang, M. (2025). Probe-based dual-chip digital loop-mediated isothermal amplification for the simultaneous detection of Staphylococcus aureus and *Salmonella* enteritidis in livestock and aquatic products. *Food Control*, 168, 110988. https://doi.org/https://doi.org/10.1016/j.foodcont.2024.110988

[24] Huoy, L., Vuth, S., Hoeng, S., Chheang, C., Yi, P., San, C., Chhim, P., Thorn, S., Ouch, B., Put, D., Aong, L., Phan, K., Nasirzadeh, L., Tieng, S., Bongcam-Rudloff, E., Sternberg-Lewerin, S., & Boqvist, S. (2024). Prevalence of *Salmonella* spp. in meat, seafood, and leafy green vegetables from local markets and vegetable farms in Phnom Penh, Cambodia [Journal Article]. *Food Microbiology*, 124, 104614. https://doi.org/https://doi.org/10.1016/j.fm.2024.104614

[25] Rheman, S., Hossain, S., Sarker, M. S., Akter, F., Khor, L., Gan, H. M., Powell, A., Card, R. M., Hounmanou, Y. M. G., Dalsgaard, A., Mohan, C. V., Bupasha, Z. B., Samad, M. A., Verner-Jeffreys, D. W., & Delamare-Deboutteville, J. (2024). Nanopore sequencing for identification and characterization of antimicrobial-resistant Escherichia coli and Salmonella spp. from tilapia and shrimp sold at wet markets in Dhaka, Bangladesh. Frontiers in microbiology, 15, 1329620. https://doi.org/10.3389/fmicb.2024.1329620

[26] Mudadu, A. G., Spanu, C., Pantoja, J. C. F., Dos Santos, M. C., De Oliveira, C. D., Salza, S., Piras, G., Uda, M. T., Virgilio, S., Giagnoni, L., Pereira, J. G., & Tedde, T. (2022). Association between Escherichia coli and *Salmonella* spp. food safety criteria in live bivalve molluscs from wholesale and retail markets. *Food Control*, 137, 108942. https://doi.org/10.1016/j.foodcont.2022.108942

[27] Yang, X., Huang, J., Su, Y., Cai, S., Zhang, J., Guo, W., Wang, J., Chen, M., Wu, S., Yang, S., & Wu, Q. (2022). Incidence and antimicrobial resistance of *Salmonella* serovars in fresh retail aquatic products from China. *Lwt*, 171, 114123. https://doi.org/https://doi.org/10.1016/j.lwt.2022.114123

[28] Akkaya, E., Muratoglu, K., Tarhan, D., Ozsobaci, N. P., Ercan, A. M., Colak, H., Hampikyan, H., Bingol, E. B., Or, M. E., Andoni, E., Ozuni, E., Gobbi, M., Petrucci, L., Di Cesare, F., Cagnardi, P., Curone, G., Balzaretti, C. M., Giaccone, V., & Castrica, M. (2025). Determination of Heavy Metal Levels and Assessment of L. monocytogenes and *Salmonella* spp. Presence in Fishery Products and Mussels from the Marmara Region, Türkiye *Toxics*. 13(3), 153. https://doi.org/10.3390/toxics13030153

[29] Sołtysiuk, M., Przyborowska, P., Wiszniewska-Łaszczych, A., & Tobolski, D. (2025). Prevalence and antimicrobial resistance profile of *Listeria* spp. isolated from raw fish. *Bmc Veterinary Research*, 21(1), 333. https://doi.org/10.1186/s12917-025-04792-y

[30] Peratikos, P., Tsitsos, A., Damianos, A., Kyritsi, M. A., Hadjichristodoulou, C., Soultos, N., & Economou, V. (2024). Listeria monocytogenes from Marine Fish and the Seafood Market Environment in Northern Greece: Prevalence, Molecular Characterization, and Antibiotic Resistance. Applied Sciences, 14(7), 2725. https://doi.org/10.3390/app14072725

[31] Gozutok, E., & Aydın, A. (2022). Presence and virulence characterization of Listeria monocytogenes from fish samples in the Black Sea, Turkey. *Ankara Üniversitesi Veteriner Fakültesi Dergisi*, 69(4), 387-394.

[32] Simonavičienė, I., Zakarienė, G., Lozoraitytė, A., Zaborskienė, G., Gerulis, G., & Stimbirys, A. (2021). Identification and serotyping of Listeria monocytogenes, isolated from various salmon products, sold in retail market in Lithuania. *Italian Journal of Food Safety*, 10(3), 9341. https://doi.org/10.4081/ijfs.2021.9341

[33] Abou Elez, R. M. M., Zahra, E. M. F., Gharieb, R. M. A., Mohamed, M. E. M., Samir, M., Saad, A. M., & Merwad, A. M. A. (2024). Resistance patterns, virulence determinants, and biofilm genes of multidrug-resistant Pseudomonas aeruginosa isolated from fish and fish handlers. *Scientific Reports*, 14(1), 24063. https://doi.org/10.1038/s41598-024-73917-4

[34] Ben Mhenni, N., Alberghini, G., Giaccone, V., Truant, A., & Catellani, P. (2023). Prevalence and Antibiotic Resistance Phenotypes of *Pseudomonas* spp. in Fresh Fish Fillets. *Foods,* 12(5), 950. https://doi.org/10.3390/foods12050950

[35] Dehkordi, S. M. H., Anvar, S. A., Rahimi, E., Ahari, H., & Ataee, M. (2022). Molecular investigation of prevalence, phenotypic and genotypic diversity, antibiotic resistance, frequency of virulence genes and genome sequencing in Pseudomonas aeruginosa strains isolated from lobster. *International Journal of Food Microbiology*, 382, 109901. https://doi.org/https://doi.org/10.1016/j.ijfoodmicro.2022.109901

[36] F. Ayoub Et Al., H. (2021). Isolation, Identification and Antimicrobial profile of *Aeromonas* spp., *Pseudomonas* spp. and *Vibrio* spp. from the Nile Tilapia, Oreochromis niloticus in fish farms. *Egyptian Journal of Aquatic Biology and Fisheries*, 25(3), 171-185. https://doi.org/10.21608/ejabf.2021.173659

[37] Algammal, A. M., Mabrok, M., Sivaramasamy, E., Youssef, F. M., Atwa, M. H., El-kholy, A. W., Hetta, H. F., & Hozzein, W. N. (2020). Emerging MDR-Pseudomonas aeruginosa in fish commonly harbor oprL and toxA virulence genes and blaTEM, blaCTX-M, and tetA antibiotic-resistance genes. *Scientific Reports*, 10(1), 15961. https://doi.org/10.1038/s41598-020-72264-4

[38] Masotti, C., Serracca, L., Costa, E., Betti, B., Garcia-Vozmediano, A., Suffredini, E., & Battistini, R. (2024). The Prevalence of Enteric Viruses in Bivalve Molluscs in a Farming Area in Liguria, Northwest Italy. *Pathogens*, 14(1), 21. https://doi.org/10.3390/pathogens14010021

[39] Cantelli, C. P., Tavares, G. C., Sarmento, S. K., Burlandy, F. M., Fumian, T. M., Maranhão, A. G., Silva, E. D., Horta, M. A., Miagostovich, M. P., Yang, Z., & Leite, J. P. (2024). Assessment of Gastroenteric Viruses in Marketed Bivalve Mollusks in the Tourist Cities of Rio de Janeiro, Brazil, 2022. *Viruses*, 16(3), 317. https://doi.org/10.3390/v16030317

[40] Bigoraj, E., Kozyra, I., Kaupke, A., Osiński, Z., Lowther, J., & Rzeżutka, A. (2024). Prevalence and quantitative assessment of foodborne viruses on the imported mussels in Polish market. *Food Control*, 157, 110145. https://doi.org/https://doi.org/10.1016/j.foodcont.2023.110145

[41] Neetoo, H., Juggoo, K., Johaheer, H., Ranghoo-Sanmukhiya, M., Manoga, Z., & Gurib, N. (2023). A study on the occurrence of human enteric viruses in salad vegetables and seafood and associated health risks for consumers in Mauritius. *Italian Journal of Food Safety*, 12(4), 11447. https://doi.org/10.4081/ijfs.2023.11447

[42] Keaveney, S., Rupnik, A., Fitzpatrick, A., Devilly, L., Fahy, J., & Doré, B. (2022). Impact of COVID-19 Nonpharmaceutical Interventions on the Extent of Norovirus Contamination in Oyster Production Areas in Ireland during Winter 2020 to 2021. *Journal of Food Protection*, 85(10), 1397-1403. https://doi.org/10.4315/JFP-22-031

[43] Pavoni, E., Bertasi, B., Galuppini, E., Mangeri, L., Meletti, F., Tilola, M., Carta, V., Todeschi, S., & Losio, M. (2022). Detection of Hepatitis A Virus and Norovirus in Different Food Categories: A 6-Year Survey in Italy. *Food and Environmental Virology*, 14(1), 69-76. https://doi.org/10.1007/s12560-021-09503-y

[44] Macaluso, G., Guercio, A., Gucciardi, F., Di Bella, S., La Rosa, G., Suffredini, E., Randazzo, W., & Purpari, G. (2021). Occurrence of Human Enteric Viruses in Shellfish along the Production and Distribution Chain in Sicily, Italy. *Foods*, 10(6), 1384 https://doi.org/10.3390/foods10061384

[45] Guarines, K. M., Mendes, R. P. G., Cordeiro, M. T., Miagostovich, M. P., Gil, L. H. V. G., Green, K. Y., & Pena, L. J. (2020). Absence of norovirus contamination in shellfish harvested and commercialized in the Northeast coast of Brazil. *Brazilian Journal of Medical and Biological Research*, 53(11), e9529. https://doi.org/10.1590/1414-431X20209529.

[46] Sarmento, S. K., Guerra, C. R., Malta, F. C., Coutinho, R., Miagostovich, M. P., & Fumian, T. M. (2020). Human norovirus detection in bivalve shellfish in Brazil and evaluation of viral infectivity using PMA treatment. *Marine Pollution Bulletin*, 157, 111315. https://doi.org/10.1016/j.marpolbul.2020.111315

[47] Das, O., Lekshmi, M., Kumar, S., & Nayak, B. B. (2020). Incidence of norovirus in tropical seafood harbouring fecal indicator bacteria. *Marine Pollution Bulletin*, 150, 110777. https://doi.org/https://doi.org/10.1016/j.marpolbul.2019.110777

[48] Suffredini, E., Le, Q. H., Di Pasquale, S., Pham, T. D., Vicenza, T., Losardo, M., To, K. A., & De Medici, D. (2020). Occurrence and molecular characterization of enteric viruses in bivalve shellfish marketed in Vietnam. *Food Control*, 108, 106828. https://doi.org/10.1016/j.foodcont.2019.106828

[49] M, A., E, R., A, S., & H, M. (2021). Incidence of hepatitis A and hepatitis E viruses and norovirus and rotavirus in fish and shrimp samples caught from the Persian Gulf. *Arquivo Brasileiro De Medicina Veterinaria E Zootecnia*, 1(73), 169-178. https://doi.org/10.1590/1678-4162-11742

[50] Bonny, P., Desdouits, M., Schaeffer, J., Garry, P., Essia Ngang, J. J., & Le Guyader, F. S. (2020). Contamination of Clams with Human Norovirus and a Novel Hepatitis A Virus in Cameroon. *Food and Environmental Virology*, 12(3), 274-277. https://doi.org/10.1007/s12560-020-09432-2

[51] NIHS. (2009). Standard Test Method for *Salmonella* spp. in Foods *NIHSJ-01-ST4*.

[52] AOAC. (2001). *Salmonella* In Selected Foods Immuno-Concentration *Salmonella* (Ics) And Selective Plate (He Bs Smid) *AOAC O.M. 2001.07*.

[53] AS. (2022). Microbiology of food and animal feeding stuffs — Horizontal method for the detection of *Salmonella* spp. *AS 5013.10:2022*.

[54] FDA. (2024). Microbiological Methods & Bacteriological Analytical Manual (BMA).

[55] ISO. (2017a). Microbiology of the food chain — Horizontal method for the detection, enumeration and serotyping of *Salmonella*. In I. T. SC (Ed.) *ISO 6579-1:2017*.

[56] NHC, & SAMR. (2024). National Food Safety Standard – Microbiological Examination of Food: Examination of *Salmonella* *GB 4789.4—2024*.

[57] ISO. (2017b). Microbiology of the food chain — Horizontal method for the determination of *Vibrio* spp. Part 1: Detection of potentially enteropathogenic *Vibrio parahaemolyticus*, *Vibrio cholerae* and *Vibrio vulnificus*. In I. T. SC (Ed.) *ISO 21872-1:2017* (33).

[58] NHFPC. (2013). National Food Safety Standard–Microbiological Examination of Food: Examination of *Vibrio parahaemolyticus* *GB 4789.7—2013* .

[59] CFDA, & NHFPC. (2016). National Food Safety Standard – Microbiological Examination of Food: Examination of *Listeria monocytogenes* *GB 4789.30-2016*.

[60] ISO. (2017c). Microbiology of the food chain — Horizontal method for the detection and enumeration of Listeria monocytogenes and of *Listeria* spp. — Part 1: Detection method. In I. TC (Ed.) *ISO 11290-1:2017*.

[61] EU-RL-FV. (2022). Standard Operating Procedure for Qualitative detection of norovirus and hepatitis A virus on surfaces *SE-751 26*.

[62] ISO. (2019). Microbiology of the food chain — Horizontal method for determination of hepatitis A virus and norovirus using real-time RT-PCR. Part 2: Method for detection. In I. T. SC (Ed.) *ISO 15216-2:2019* (40).
